# Effects of Internet Popular Opinion Leaders (iPOL) Among Internet-Using Men Who Have Sex With Men

**DOI:** 10.2196/jmir.2264

**Published:** 2013-02-25

**Authors:** Nai-Ying Ko, Chao-Hsien Hsieh, Ming-Chi Wang, Chiang Lee, Chun-Lin Chen, An-Chun Chung, Su-Ting Hsu

**Affiliations:** ^1^Department of NursingCollege of MedicineNational Cheng Kung University and HospitalTainanTaiwan; ^2^Departments of Computer Science and Information EngineeringNational Cheng Kung UniversityTainanTaiwan; ^3^Sunshine Queer CenterTaiwan Love and Hope Association,KaohsiungTaiwan; ^4^Department of PsychiatryCollege of MedicineKaohsiung Chang Gung Memorial Hospital and Chang Gung UniversityKaohsiungTaiwan

**Keywords:** Internet, intervention studies, HIV prevention, Homosexuality, Male

## Abstract

**Background:**

The Internet has become a popular venue for facilitating sex networking for men who have sex with men (MSM).

**Objective:**

The study aimed to evaluate the effectiveness of Internet popular opinion leaders (iPOL) in disseminating information about the human immunodeficiency virus (HIV), increasing the frequency of HIV testing, and reducing risky behaviors among MSM in Taiwan.

**Methods:**

A quasi-experimental study with a nonequivalent control website for comparison was used to estimate the effectiveness of iPOL. A community-level intervention, iPOL, was conducted at the Facebook social networking website and at top1069 as a control. The iPOLs actively disseminated HIV-related information via the platform of Internet opinion leaders and AIDS information center, and discussed and responded to questions or replied to Internet-using MSM.

**Results:**

A total of 369 iPOLs posted 432 articles and 503 replies to others, influencing 959,088 persons on Facebook. A total of 1037 MSM, 552 (53.23%) from an intervention website and 485 (46.76%) from a control website, participated in the follow-up study survey (response rate 96%). At the 6-month follow-up after the intervention was conducted, MSM who visited the intervention website were more likely to receive HIV-related information (25.49% versus 10.47%, *P*<.001), discuss HIV issues with others (41.88% versus 23.79%, *P*<.001), review articles about HIV (90.58% versus 79.73%, *P*<.001), and be asked about or discuss HIV-related questions (51.11% versus 31.78%, *P*<.001) than those on the control website. In addition, MSM were more likely to have HIV tests within 6 months (43.89% versus 22.31%, *P*<.001) and consistently use condoms during anal sex with online sex partners than those using the control website (34.15% versus 26.19%, *P*=.004).

**Conclusions:**

The study showed the feasibility and effectiveness of the iPOL intervention as an online HIV prevention program. These findings underscore the importance of disseminating HIV information online, as well as the challenges inherent in the efforts of iPOL to reduce HIV-related risky behaviors among Internet-using MSM.

## Introduction

Finding male sex partners through the Internet has been associated with human immunodeficiency virus (HIV) and syphilis outbreaks among men who have sex with men (MSM) who meet online [1,2]. The Internet has become a significant venue for meeting MSM, who then engage in risky behaviors associated with HIV and sexually transmitted infection (STI) transmission [3,4]. The Asia Internet MSM Sex Survey in Taiwan reported that 73.9% of MSM had had sex with partners found online and that having sex with online partners was associated with a history of STIs, recreational drug use, and an increasing number of sexual partners [5]. The Internet has become a popular meeting place for MSM; thus, effective and targeted prevention programs should be developed to minimize the HIV transmission risk in the Internet era [6].

Information and communications technology has the potential to improve the quality and efficiency in HIV/STI prevention. The social network site, Facebook, has gained enormous popularity throughout the world, has created a mechanism for acquainting support from friends with same diseases [7], and provides a promising medium to deliver HIV/STI prevention messages to Internet users [8]. Online social network usage and the topics discussed on these networks were associated with HIV knowledge and risky sexual behaviors, and testing for STIs among homeless adolescents [9]. Homeless youth who used social networking websites, such as MySpace and Facebook, to connect with family members online were less likely to practice exchanged sex and more likely to report a recent HIV test, whereas youth connected to street-based peers online were more likely to practice exchanged sex [10]. The UCLA Harnessing Online Peer Education (HOPE) study used Facebook to scale the community popular opinion leader (C-POL) model to increase HIV prevention among African American and Latino MSM [11]. The Just/Us study aims to engage youth of color in sexual health education delivered via Facebook [12]. However, the findings of a few intervention studies that used Facebook as a medium for HIV prevention have not been reported yet. Because social media plays a more prominent role in the delivery of HIV/STI prevention intervention, more Internet-based HIV prevention research is required, particularly with changing behaviors among populations at high risk of contracting HIV/AIDS.

The Internet popular opinion leaders (iPOL) intervention for HIV/STI prevention was first designed in 2010 on Facebook for online Chinese MSM communities. The iPOL intervention was adapted from the popular opinion leader (POL) model, developed by Kelly in 1986 [13] and based on the diffusion of innovation theory [14]. The POL model posits that behavioral change is achieved when new risk-reducing methods for HIV prevention are disseminated by opinion leaders through their personal and social networks [15]. POL uses ethnographic techniques to systematically identify popular and socially influential members of the target population, then recruits and trains these popular individuals in how to communicate HIV risk reduction endorsement messages to peers during everyday conversations, and works with them to sustain their HIV prevention advocacy activities [16].

Computer technology-based HIV prevention interventions have been effective in decreasing the frequency of sexual behavior, the number of partners, and incidences of sexually transmitted diseases with an efficacy similar to more traditional human-delivered interventions [17]. Different from previous 1-way technology-based HIV prevention interventions [17], the iPOL is one type of community-level intervention using the Web 2.0 2-way communication format on Facebook to deliver HIV prevention messages for Internet-using MSM. Social networking websites, such as Facebook, hold great potential in adapting the POL intervention model on the Internet to diffusing HIV-related information among Internet-using MSM. MSM with a high degree of opinion leadership in the iPOL intervention are identified within existing online social networks and then trained in persuasive techniques and prevention messages to shape the behavior of their affiliated online network members [18].

Social networks provide a promising mechanism to deliver HIV prevention messages among Internet-using MSM. The Internet provides a more convenient approach to allowing MSM to answer questions anonymously and, therefore, maintain their privacy [19]. This study is the first Internet-based HIV intervention study in Taiwan to determine the effectiveness of iPOL in disseminating HIV-related information, increasing the frequency of anonymous HIV testing, and reducing risky behaviors among Internet-using MSM.

## Methods

### Study Design

A quasi-experimental study with a nonequivalent control website for comparison was used to estimate the effectiveness of iPOL among the Internet-using MSM population. The iPOL intervention was conducted from April through September 2011 at the Facebook social networking website and the top1069 website as a comparison.

### Recruitment and Consent Procedures

Participants were recruited exclusively through online methods, primarily through Web banners on the top1069 website and electronic direct mailers sent through a network of gay community coalition partners on Facebook. Informed consent was requested from all participants on the first page of the questionnaire, and only those participants who said they were at least 18 years old and had had sex with a man in the past 12 months were given access. Approval to conduct the study was obtained from the Human Subjects Division Committee of National Cheng Kung University Hospital.

A cross-sectional online survey was used to collect baseline data of HIV-related behaviors at the intervention and comparison websites from October to November 2010. After the iPOL intervention had been implemented for 6 months, the same online survey was conducted from October to November 2011. During the baseline survey period, 2042 participants entered the survey. Of the 1692 (82.85%) participants who completed the baseline online survey, 1008 provided valid information for further analysis. After the 6-month intervention, 1079 men completed the follow-up online survey, excluding those aged less than 18 years (n=32) and transgender individuals (n=10). A total of 1037 MSM, 552 (53.23%) from the intervention website and 485 (46.76%) from the control website, participated in the follow-up study survey.

### Internet Popular Opinion Leaders Intervention

An online ethnographic study was carried out in virtual MSM communities in Taiwan. A series of Internet searches were conducted from January through March 2010 using 3 standard search engines (Google, Yahoo, and Ping), querying for traditional Chinese translations of terms including gay, homosexuals, AIDS, HIV, HIV and sexually transmitted diseases, and other relevant search terms of interest. We identified each online virtual MSM community based on interaction between MSM online users, electronic contents and replies posted within the community, and HIV/AIDS-related information dissemination serving the needs of venue MSM members. The iPOLs were recruited and recommended by networks of gay community coalition partners. The recommended eligible iPOLs were assessed by 6 questions about their opinion leadership [20] on HIV/STI information in the previous 3 months, including how they talked to their friends about HIV/STIs, how they gave information on HIV/STIs to their friends, how many people they told about HIV/STIs, how likely they were to be asked about HIV/STIs, and how they would respond to their friends about HIV/STIs. A total of 369 men with a high degree of opinion leadership were selected as iPOL.

**Figure 1:**
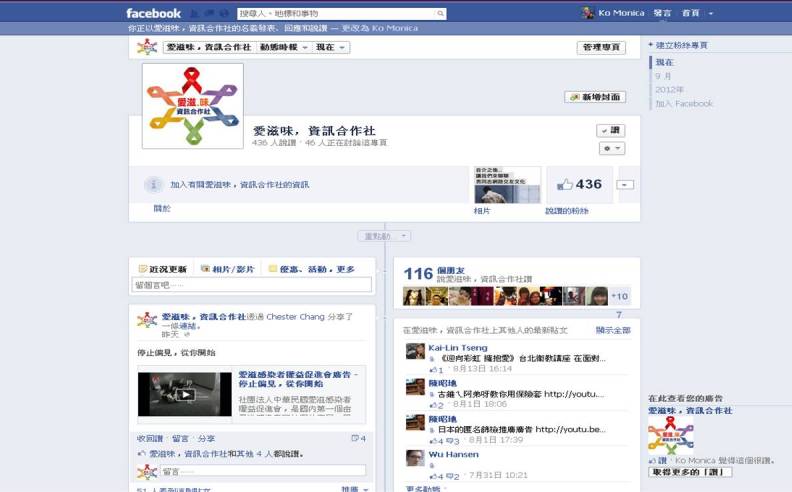
Sample iPOL platform on Facebook in 2012.

The 369 iPOL were trained by HIV/STI experts in the fundamentals of HIV prevention, social marketing strategies for dissemination of innovative ideas, and strategies of risk reduction and behavior change during a 12-week period in 2010. The online iPOL platform built on Facebook (Figure 1) was used to scale-up the influence of iPOLs on HIV prevention among Internet-using MSM. The iPOL platform uses the Web 2.0 2-way communication format on Facebook in which the iPOLs share and exchange news, video clips, reports, and opinions, and have the capability to connect with others over the Internet for advice and support. During the iPOL intervention period from April through September 2011, 2-way conversations related to risky behaviors on the online iPOL platform were reviewed by iPOLs, discussed, and reinforced at subsequent iPOL training sessions through the end of 2011. By the end of 2011, there were approximately 432 posts (including film clips, news, videos, personal accounts, and discussions on risky behavior), 503 comments, and 804 likes on the iPOL platform, with an estimated 959,088 people viewing the posts on the iPOL platform.

### Measures

The online survey included demographic information on sex, age, residency, ethnicity, employment, education (primary or no formal education, secondary, tertiary, professional qualification, university, and postgraduate degree), partnership status (never married, opposite sex marriage, same sex marriage or civil partnership, divorced, separated, and widowed), and sexual orientation. Participants were first asked whether they had heard about iPOL or the AIDS information center, followed by a series of questions about experiences discussing HIV-related issues with online friends, reading HIV-related posts on the Internet, and having online friends talk to them about HIV-related issues. They were asked about their HIV test history (date and result of the last test), recreational drug use, history of STIs, and sexual behavior in the previous 3 months, including sexual behavior with males and females, the number of male and female sexual partners, sexual behavior with online partners, condom use behavior, unprotected anal intercourse (UAI), and use of the Internet to look for sex.

### Analysis

Statistical analyses were performed with SPSS version 17.0 for Windows software (SPSS Inc, Chicago, IL, USA). HIV information distribution, HIV testing behavior, and risky behaviors were the main intervention outcomes. Intervention effects were assessed 6 months after the intervention. Between-group differences for primary behavior outcomes among Internet-using MSM were measured using chi-square tests at the 6-month follow-up. Mixed-effects logistic regression analysis, controlling for the baseline, was used to examine changes in HIV information dissemination, HIV testing behavior, and risky behaviors between the intervention and comparison websites.

## Results

### Characteristics of the Study Participants

A total of 1037 men were recruited online, 499 (48.12%) from the intervention website and 538 (51.88%) from the comparison website. Significant differences in demographic characteristics were found between the comparison and treatment groups (Table 1). These differences were in age (men in the comparison group were younger), partnership status (a greater percentage of men in the comparison group were single), and education (those in the intervention group had slightly more years of education). Behavioral characteristics between the men in the comparison and intervention websites were similar, except that men enrolled in the intervention website were more likely to use recreational drugs, been diagnosed with STIs, and be HIV positive than those in the comparison website.

**Table 1:** Comparison of demographic and health characteristics between participants in the comparison and intervention (iPOL on Facebook) groups (N=1037).

Demographic and health characteristics		Total^a^	Intervention^a^ n=499	Comparison^a^ n=538	*P* value
Age (years), mean (SD)		24.8 (6.2)	25.8 (6.3)	23.8 (5.9)	<.001
Residency, n (%)					
	North	476 (45.90)	231 (46.29)	245 (45.53)	.007
	Middle	179 (17.26)	67 (13.42)	112 (20.81)	
	South	345 (33.26)	184 (36.87)	161 (29.92)	
	East/Archipelagos	37 (3.56)	17 (3.40)	20 (3.71)	
**Education level, n (%)**					<.001
	High school or less	15 (1.44)	5 (1.00)	10 (1.85)	
	Some college	214 (20.63)	70 (14.02)	144 (26.76)	
	College	656 (63.25)	324 (64.92)	331 (61.71)	
	Postcollege	152 (14.65)	100 (20.04)	52 (9.66)	
Partnership status, n (%)					<.001
	Single	601 (57.95)	246 (49.29)	355 (65.98)	
	Married (opposite sex)	11 (1.06)	8 (1.60)	3 (0.55)	
	Civil partnership (same sex)	388 (37.41)	228 (45.69)	160 (29.73)	
	Steady sex partner	37 (3.56)	17 (3.40)	20 (3.71)	
**Sexual behavior, n (%)**					
	Had sex with females in the previous 3 months	39 (3.76)	17 (3.40)	22 (4.08)	.63
	Sought sex on the Internet in the previous 3 months	765 (73.77)	361 (72.34)	404 (75.09)	.32
	Had online sex partners in the previous 3 months	543 (52.36)	245 (49.09)	298 (55.39)	.046
	Consumed recreational drugs in the previous 3 months	206 (19.86)	121 (24.24)	85 (15.79)	.001
	Diagnosed with STIs in the previous 3 months	59 (5.68)	40 (8.01)	19 (3.53)	.002
	HIV positive	51 (4.91)	40 (8.01)	11 (2.04)	<.001

^a^ May not add to total because of missing responses.

### Effects of Internet Popular Opinion Leaders on Dissemination of HIV-Related Information

The iPOL intervention had significant effects on the dissemination of HIV-related information (Table 2). We found there was no difference in HIV information distribution between the comparison and intervention groups at baseline. At the 6-month follow-up after controlling for baseline data, participants that visited the intervention website were more likely to access HIV-related information from iPOL or the AIDS information center (25.49% versus 10.47%, *P*<.001), to discuss HIV-related issues with online friends (41.88% versus 23.79%, *P*<.001), to read HIV-related posts on the Internet (90.58% versus 79.73%, *P*<.001), and to have online friends talking about HIV-related issues (51.11% versus 31.78%, *P*<.001) than those in the comparison group.

**Table 2:** Intervention effects on HIV information distribution among Internet-using MSM.

Outcome variables		Baseline^a^, n (%) n=1008		Follow-up, n (%) n=1037		χ^2^_1_	*P* value
		Intervention^b^ n=501	Comparison^b^ n=507	Intervention^b^ n=499	Comparison^b^ n=538		
Ever heard about iPOL or the AIDS information center on Facebook?							
	No	N/A	N/A	269 (74.51)	453 (89.52)	34.1	<.001
	Yes	N/A	N/A	92 (25.49)	53 (10.47)		
Discussed HIV-related issues with online friends							
	No	444 (88.62)	461 (90.92)	290 (58.11)	410 (76.20)	38.6	<.001
	Yes	57 (11.37)	46 (9.07)	209 (41.88)	128 (23.79)		
Read HIV-related posts on the Internet							
	No	413 (82.43)	431 (85.01)	47 (9.41)	109 (20.26)	23.8	<. 001
	Yes	88 (17.56)	76 (14.99)	452 (90.58)	429 (79.73)		
Online friends talked to you about HIV-related issues							
	No	413 (82.43)	437 (86.19)	244 (48.89)	367 (68.22)	39.9	<. 001
	Yes	88 (17.57)	70 (13.81)	255 (51.11)	171 (31.78)		

^a^ There is no difference in HIV information distribution at baseline between comparison and intervention groups.

^b^ May not add to total because of missing responses.

### Effects of Internet Popular Opinion Leaders on HIV Testing and Sexual Behaviors

The iPOL intervention also had partial effects on HIV-related behavior (Table 3). At the 6-month follow-up after controlling for baseline data, participants that visited the intervention website were more likely to have had HIV tests in the past 6 months (43.89% versus 22.31%, *P*<.001) and to use a condom during anal sex with online sex partners compared to the control website (34.15% vs 26.19%, *P*=.004). However, there was no difference in the number of male online anal sex partners, the number of male partners with UAI, and condom use during anal sex with male sex partners between MSMs visiting the intervention website and those from comparison website.

**Table 3:** Intervention effects on HIV testing and sexual behaviors within the previous 3 months among Internet-using MSM.

Outcome variables		Baseline^a^, n (%) n=1008		Follow-up, n (%) n=1037		χ^2^ (df)	*P* value
		Intervention^b^ n=501	Comparison^b^ n=507	Intervention^b^ n=499	Comparison^b^ n=538		
Had HIV test in the past 6 months							
	No	351 (70.05)	360 (71.14)	280 (56.11)	418 (77.69)	54.8 (1)	.001
	Yes	150 (29.95)	146 (28.86)	219 (43.89)	120 (22.31)		
**Number of male sexual partners**							
	0	145 (29.00)	145 (28.71)	117 (23.44)	175 (32.52)	9.9 (3)	.02
	1	182 (36.40)	176 (34.85)	170 (34.06)	163 (30.29)		
	2-5	145 (29.00)	155 (30.69)	176 (35.27)	172 (31.97)		
	≥ 6	28 (5.60)	29 (5.74)	36 (7.21)	28 (5.20)		
Number of male online sex partners							
	0	283 (56.48)	267 (52.87)	256 (51.30)	247 (45.91)	7.6 (3)	.06
	1	85 (16.96)	104 (20.59)	72 (14.42)	112 (20.81)		
	2-5	115 (22.95)	112 (22.17)	148 (29.69)	155 (28.81)		
	≥ 6	18 (3.59)	22 (4.43)	23 (4.60)	24 (4.47)		
Number of male partners with unprotected anal sex							
	0	295 (59.23)	302 (60.15)	279 (55.92)	308 (57.25)	2.3 (3)	.52
	1	143 (28.71)	142 (28.28)	144 (28.85)	158 (29.36)		
	2-5	54 (10.84)	52 (10.35)	66 (13.22)	67 (12.45)		
	≥6	6 (1.20)	6 (1.19)	10 (2.01)	5 (0.93)		
Condom use during anal sex with online sex partners							
	Never	154 (49.67)	160 (48.63)	92 (32.39)	133 (39.58)	13.4 (3)	.004
	Sometimes	49 (15.80)	47 (14.28)	41 (14.43)	72 (21.43)		
	Most of the time	39 (12.59)	37 (11.24)	54 (19.01)	43 (12.80)		
	All the time	68 (21.93)	85 (25.83)	97 (34.15)	88 (26.19)		
Condom use during anal sex with male sex partners							
	Never	92 (25.92)	100 (27.85)	81 (24.92)	92 (24.79)	3.0 (3)	.39
	Sometimes	75 (21.12)	68 (18.94)	68 (20.92)	64 (17.25)		
	Most of the time	65 (18.31)	60 (16.71)	54 (16.62)	78 (21.24)		
	All the time	123 (34.65)	131 (36.49)	122 (37.54)	137 (36.91)		

^a^ There is no difference in HIV testing and sexual behaviors at baseline between comparison and intervention groups.

^b^ May not add to total because of missing responses.

## Discussion

The current study extended prior HIV prevention research by adapting the C-POL model to the Internet. Our study created an iPOL platform on Facebook for dissemination of HIV prevention messages by 369 trained POLs on the Internet, and an estimated 959,088 people viewed the posts on the iPOL platform within 6 months. The findings of the current study are consistent with the results of social influence analysis of Facebook users, in that influential people with influential friends help spread information [21]. Over 70% of the MSM recruited online in Taiwan sought sex partners online [22], indicating that the Internet is an important venue for conducting HIV prevention programs for MSM [4,22]. Social networks are a useful tool for supporting people affected by HIV infection and patients suffering from HIV disease [23]. In terms of using these social networks for disease support purposes, Facebook shows a higher usage rate than Twitter [24]. Our study demonstrates that the use of iPOL on Facebook to disseminate HIV-related information to MSM is both promising and practical. The networking capability and participatory and interactive features of Facebook can be used to foster solidarity and deepen the involvement of Internet-using MSM in HIV prevention.

To our knowledge, this is the first study to measure the effects of applying the POL intervention model to the Internet. Our study shows the significant effects of the iPOL intervention on stimulating conversation about HIV among MSM on social networks and increasing the use of HIV testing. Consistent with a previous study in coastal Peru on the effects of a C-POL HIV/STI intervention on stigma [25], our study showed that iPOL intervention can significantly increase the dissemination of HIV-related information and stimulate conversation about HIV-related risky behaviors in online MSM communities. Multimedia social marketing campaigns have had a significant impact on HIV testing uptake [26]. Our study results support the role of iPOL intervention in disseminating HIV information online that could potentially increase rates of HIV testing, consistent with similar findings that online social network usage is associated with increasing HIV testing among homeless youth [9]. In the United States, health agencies such as the Centers for Disease Control and Prevention Twitter Chat and AIDS.gov have begun using online social networks to inform users about their STI risks and the services available to them [27]. Using influential online social networking to increase the number of conversations about the resources of HIV testing, HIV-related risks and prevention behaviors can increase awareness of HIV prevention among Internet-using MSM. Further studies are needed to develop online HIV intervention with multiple-language support, evaluate the accuracy of online HIV information, and document the gaps that exist when searching for information online.

The iPOL intervention showed limited effects in reducing HIV risky behaviors among Internet-using MSM. The iPOL intervention can increase condom usage with online sex partners, but there was no significant effect on reducing the number of online male sex partners and UAI partners or in increasing condom use with male sex partners. Our study results were consistent with those of previous studies that demonstrated that the C-POL intervention for homosexual men in US cities was effective in increasing condom use during anal intercourse [28-30], but inconsistent with the C-POL intervention in decreasing UAI [28,29]. A possible explanation for this is that the effective interventions for reducing the number of sexually risky behaviors of MSM emphasized interpersonal skills training and incorporated several delivery methods [31], which cannot be achieved by increasing the dissemination of HIV information alone. It is possible that in addition to creating a platform to exchange HIV information, the iPOL intervention also increased the possibility of using online social networks to become acquainted with new friends [32] and seeking potential sex partners. These findings underscore the importance of disseminating HIV-related information online, as well as the challenges inherent in the efforts of iPOL to influence norm-changing approaches and reduce HIV-related risky behavior among Internet-using MSM.

Several limitations of this study should be noted. First, because it was difficult to randomize Internet users to either the intervention or the control website, this quasi-experimental study design with a nonequivalent control group for comparison was especially susceptible to the threats of cross-contamination on the effects of iPOLs. To reduce the differences in outcome variables that existed between groups exposed to the intervention, participants in the control group who reported using Facebook as their website for seeking sex partners were excluded. Second, selection bias resulting from a convenient cross-sectional design may limit the generalizability of the research findings. It is difficult to determine whether the study participants represented the MSM population as a whole or only those MSM using the Internet. Third, data on HIV testing and sexual behaviors were self-reported, which is a limitation of many behavioral studies. Although the study was conducted anonymously through the Internet, there could still be underreporting of sensitive questions, such as HIV status, UAI, or illicit substance use.

In conclusion, an iPOL platform on Facebook was built for dissemination of HIV prevention messages by 369 trained POLs, with an estimated 959,088 people viewing the posts within the 6-month period of intervention. The iPOL intervention had significant effects on the dissemination of HIV-related information and on increasing the frequency of HIV testing among Internet-using MSM. However, the iPOL intervention had a limited impact on individual risky sexual behaviors. Adaptation of the POL model to the Internet to stimulate conversation about HIV and increase HIV testing among MSM is both effective and promising.

## Abbreviations

C-POL: community popular opinion leader
HIV: human immunodeficiency virus
iPOL: Internet popular opinion leader
MSM: men who have sex with men
POL: popular opinion leader
STI: sexually transmitted infection
UAI: unprotected anal intercourse
